# Computed tomography angiography of a congenital extrahepatic splenocaval shunt in a foal

**DOI:** 10.1186/s13028-019-0474-0

**Published:** 2019-08-14

**Authors:** Dorien Suzanne Willems, Lieuwke Cecilia Kranenburg, Josina Margaretha Ensink, Anne Kummeling, Inge Dagmar Wijnberg, Stefanie Veraa

**Affiliations:** 1 Division of Diagnostic Imaging, Faculty of Veterinary Medicine, Utrecht University, Utrecht, The Netherlands; 2 Department of Equine Sciences, Faculty of Veterinary Medicine, Utrecht University, Utrecht, The Netherlands; 3 Department of Clinical Sciences of Companion Animals, Faculty of Veterinary Medicine, Utrecht University, Utrecht, The Netherlands

**Keywords:** Equine, Hepatic, Horse, Imaging, Liver, Portosystemic shunt

## Abstract

Congenital portosystemic shunts in foals are rare and only a small number of cases have been described. Detailed description of the course of the shunt is lacking in earlier reports. This is the first detailed description of a computed tomography angiography (CTA) displaying an extra-hepatic splenocaval shunt. A 1-month old colt showing increasing signs of dullness, ataxia, circling, lip-smacking and coordination problems was presented. Hyperammonemia was detected and abdominal CTA revealed an extra-hepatic portocaval shunt. During surgery, ligation of the abnormal vessel could not be achieved, and the foal was euthanized because of complications during surgery. CTA provided a detailed overview of portal vasculature. If a portosystemic shunt is suspected in a foal, CTA can be used to confirm the diagnosis and for surgical planning.

## Background

A portosystemic shunt is an abnormal vessel between the portal venous system and the systemic circulation, bypassing the liver [1, 2]. Congenital portosystemic shunts can be intra- or extrahepatic. Congenital portosystemic shunts have frequently been reported in small animals [1, 2] but are considered rare in foals. In the last 25 years only four cases of portosystemic shunts in foals have been described [3–5]. In two of those cases the shunt was extrahepatic. These foals presented with dullness, intermittent neurological signs, hypersalivation, blindness and disorientation. Hyperammonemia was found in all cases, which makes hepatic encephalopathy a likely cause of the clinical signs and imaging of the portal vein was attempted. The imaging techniques used to explore the course of the shunt included ultrasonography, positive contrast portography and computed tomography angiography (CTA) (Table 1). Unfortunately, the description of the course of the shunts is limited in these reports. CTA was only used in one case of an intrahepatic shunt [4]. This is the first detailed description of the course of an extra-hepatic splenocaval shunt explored using CTA.

**Table 1:** Documented porto-systemic shunts in foals

	Clinical signs	Laboratory values	Imaging findings	Treatment	Outcome	Ref.
Belgian, male age 5 weeks	Episodic disorientation, recumbency, thrashing, nonresponsive to external auditory stimuli, apparently blind and would throw his head and lunge violently	Blood ammonia 380/40 μmol/L (sample/control) Total bile acids 86 μmol/L (< 20) Total bilirubin 12.0 mg/dL (0.3–3.5) γGT 21 U/L (10–59) BUN 42 mg/dL (10–27)	Ultrasound: large vessel appeared to communicate with the caudal vena cava Positive contrast portography: intrahepatic portocaval shunt outlined by contrast agent flowing from the portal vein to the caudal vena cava without parenchymal perfusion	Surgery: shunt ligated with 2 polypropylene	Shunt ligation loosened within 16 days Attempts to relegate led to uncontrollable hemorrhages Euthanasia was performed	[5]
Belgian, female age 5 months	Small for her breed and age, clumsiness, acute blindness, head pressing, circling, staggering, circling, dragging all four feet, no proprioceptive deficits, apparently blind	Blood ammonia 179/21 μmol/L (sample/control) Total bile acids 83 μmol/L (< 20) Total bilirubin 1.7 mg/dL (0.3–3.5) γGT 29 U/L (10–59) BUN 11 mg/dL (10–27)	Positive contrast portography: large portocaval shunt delineated by contrast agent. No filling of portal veins within the liver was observed	Surgery: shunt ligated with double ligatures of 5 polyester suture material	Clinical improvement. Persistent increased blood ammonia (100 μmol/L) and serum bile acids (24 μmol/L) The foal remained healthy, without recurrence of clinical signs 2 years after ligation, but stayed small for her breed	[5]
Mixed breed Arab, female age 9 weeks	Intermittent episodes of circling, incoordination, absence of menace reflex, apparent blindness, inability to nurse, lethargy, unresponsiveness, ptyalism, bruxism, mild apathy, circling, ataxia, hypermetric gait of the forelimbs, and high head carriage	Blood ammonia 208 μmol/L (< 50) Total bilirubin 4.8 mg/dL (0.5–2.3)	Ultrasound: no shunt vessel identified CT angiography: an abnormal vessel originated from the intrahepatic portion of the portal vein, entering the most ventral aspect of the caudal vena cava immediately caudal to the diaphragm Echocardiography with transsplenic injection of agitated saline: immediate after injection contrast in the right atrium and ventricle Intra-operative ultrasound: Intrahepatic portocaval shunt identified 2 cm inside the liver parenchyma, running parallel and in contact with the caudal vena cava and entering the most ventral aspect of the caudal vena cava, immediately caudal to the diaphragm	Surgery: shunt ligated with cellophane	Clinical improvement within 2 days Blood ammonia still slightly increased 6 weeks after surgery (54 μmol/L), bile acid normal (5 μmol/L) After 7 months: foal is bright and alert	[4]
American miniature, female age 5 weeks	Hypersalivation, trismus, poor appetite, hyperaesthesia, aimless wandering and blindness Developed generalized pruritus, head pressing, marked ataxia, hindlimb stiffness, hypermetria, ataxia in all 4 limbs, disorientation, walking into stationary objects, difficulties locating the dam, no menace reflex and the pupillary light reflex absent bilaterally	Blood ammonia 92.0 μmol/L (7.6–63.2 μmol/L) Bile acid 54.6 μmol/L (< 15 μmol/L) γGT 86 iu/l (slightly elevated) Creatinine 53 μmol/L (decreased) BUN 1.8 mmol/L (decreased)	Ultrasound: liver reduced in size, hepatic portal vein and caudal vena cava were identified. An abnormal vessel arised from the prehepatic portal vein, which looped dorsally and caudally to merge with the caudal vena cava near the right renal vein Intra-operative mesenteric portovenography: a single extrahepatic PSS was identified curving dorsally into the caudal vena cava Cranial to the origin of the shunt, the portal vein was markedly reduced in diameter	Surgery: shunt ligated with 4 metric silk	Clinical improvement. The foal had grown to normal size and had shown no clinical disease At 3 years of age subjected to euthanasia due to severe abdominal pain. Post mortem examination was not performed	[3]
Dutch Warmblood, male age 2 months	Episodes of apathy and ataxia, gnashing, circling, apparent blindness, depression, hypermetria alternated with dysmetria of all 4 limbs, bilateral horizontal nystagmus, ptosis, variable menace reflex and delayed correction reflexes	Blood ammonia 117 µmol/L (11–55) Bile acid 53 μmol/L (1–8.6) Unconjugated bilirubin 168 μmol/L (< 35)	CT angiography: abnormal vessel identified, looping from the portal vein to the caudal vena cava at the region. The abnormal vessel looped to the left and caudally, entering the left side of the caudal vena cava, just cranial to the left renal vein. The total length of the shunting vessel was approximately 5 cm. Cranial to the shunt, a well-developed portal vein continued, the gastroduodenal vein joined the portal vein and the vein branched into the liver	Surgery: cellophane ligation was planned. Surgery aborted due to complications	Euthanasia	–

## Case presentation

A 1-month old Dutch Warmblood colt was presented to the Equine Clinic at Utrecht University (NL) during emergency hours with episodic neurological signs. The neurological signs were first noted after the first day at pasture, at 1 week of age, and included mild ataxia, circling, lip-smacking and biting at the right carpus. During this first visit to the clinic intermittent very mild, but similar, abnormalities were observed at general and neurological clinical examination. Blood chemistry and hematology on admission revealed mild leucocytosis (12.8 × 10^9^/L, reference range 4.7–10.0), an increased lactate concentration (4.2 mmol/L, reference range 0.7–1.2) and low gamma-immunoglobulins (2.7 g/L, reference range 6–19). Trauma could not be excluded and the foal was treated with equine hyperimmune plasma (Hypermune),^1^ flunixin meglumine (Finadyne)^2^ and vitamin E (Equi-vitamin E).^3^ The neurological signs resolved during the initial few hours of hospitalisation and the foal was discharged from the clinic after 48 h, appearing bright and alert, but less lively than might be expected given his age.

Episodic neurological signs were noted by the owner during the following weeks and became more severe, when present. Upon the second admittance to the clinic, 4 weeks later, the foal was dull and demonstrated ataxia, bruxism, circling, kept his head low and appeared to be blind. He had trouble localizing the udder to drink and there were severe bite marks on the teats of the dam. Clinical findings that were noted on repeated neurological examination included intermittent dullness, hypermetria alternated with dysmetria of all four limbs, intermittent bilateral horizontal nystagmus, ptosis, variable presence of the menace response and delayed postural reactions. Clinical signs were suspicious of a metabolic disorder based on the variability of severity, occurrence and suspected origin of deficits. Further diagnostics revealed hyperammonemia (117 µmol/L, reference range 11–55), increased bile acids (53 μmol/L, reference range 1–8.6), unconjugated bilirubinemia (168 μmol/L, reference < 35), and leucocytosis (14.3 × 10^9^/L, reference range 4.7–10). A congenital portosystemic shunt was suspected.

CTA of the foal’s abdomen was performed to confirm the presence and determine the location of the portosystemic shunt. The foal was premedicated with 0.2 mg fentanyl (Fentadon)^4^ and 10 mg midazolam (Midazolam Actavis)^5^ IV, and anesthesia was induced with 350 mg propofol (Propofol)^6^ IV. The foal was intubated and general anesthesia maintained by isoflurane inhalation (ET isoflurane 1.2%). The animal was positioned in dorsal recumbency on the patient table of a 64-slice CT Scanner (Siemens Somatom Definition AS sliding gantry). CTA included an unenhanced scan and enhanced scans. Enhanced scans were available for the early venous phase and late venous phase. The contrast medium used in this study was iobitridol (Xenetix).^7^ One minute after manual intravenous bolus injection of 100 mL of contrast medium (1 mL/kg) via the jugular vein was completed, the early venous phase was manually started and the foal was scanned from the diaphragm to the mid abdomen. A late venous phase scan of the entire abdomen was initiated 1 min after the previous scan. Scanning direction was from cranial to caudal in both scans. The following technical parameters were used: 120 kV, 340 mAs, 3 mm slices, 0.5 s tube rotation, pitch 0.9, helical acquisition. Images were reconstructed using a soft tissue algorithm (B30F) with a matrix size of 512 × 512 and reconstruction Field Of View of 442 × 442 mm.

**Fig. 1:**
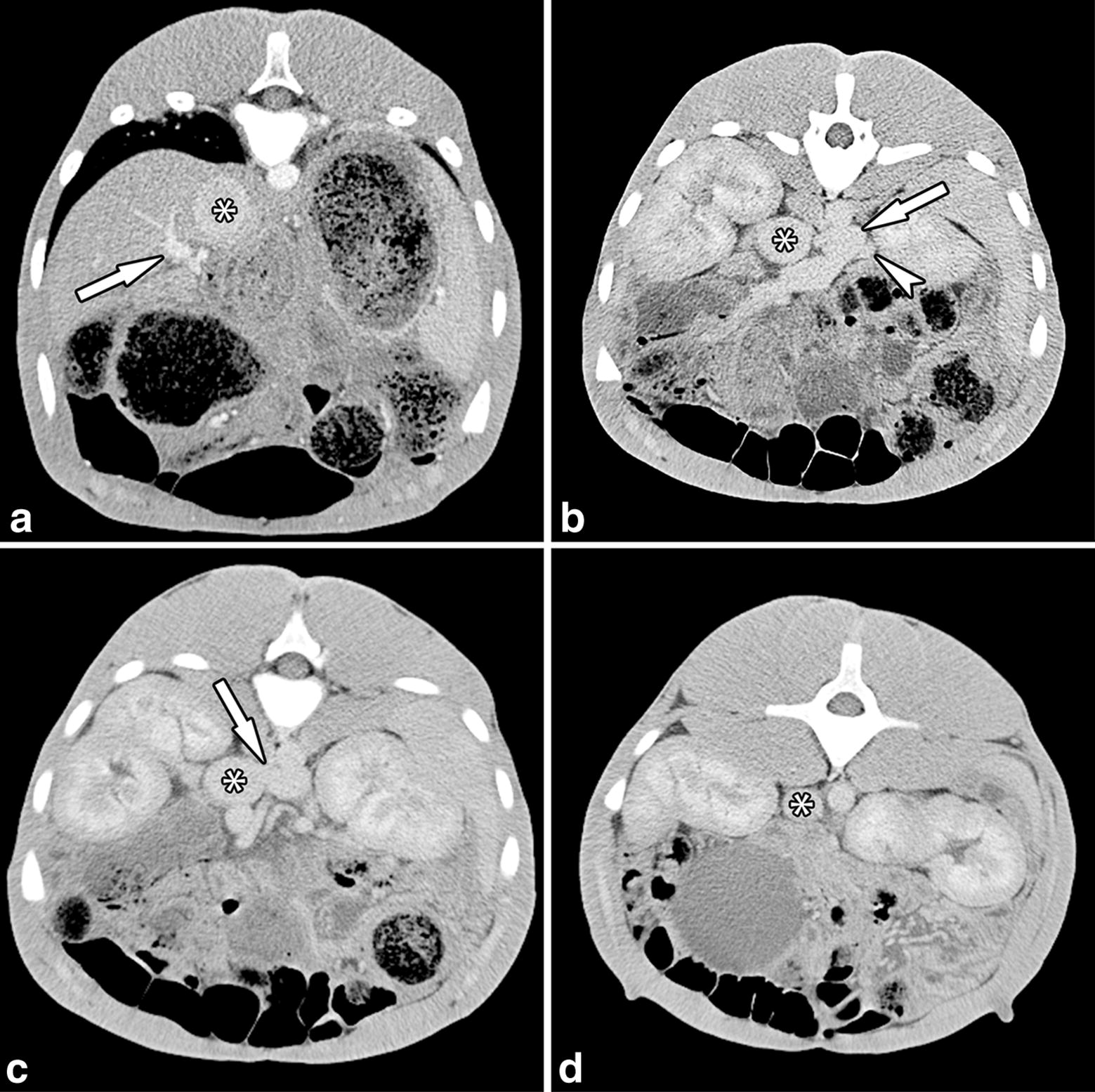
Transverse computed tomographic images of the foal with a single extrahepatic portocaval shunt. The letters (**a**, **b**, **c** and **d**) denote the right side of the patient and **a** to **d** is cranial to caudal. **a** Cranial to the shunt the remaining portal vein (arrow) enters the liver and the caudal vena cava (asterisk) appears normal in size, shape and position. **b** A broad and short abnormal vessel arises from the combined caudal and cranial mesenteric veins (arrow), at the level of the junction of the splenic vein (arrowhead) and the mesenteric veins. **c** The shunt (arrow) merges with the caudal vena cava (asterisk) on the left side. **d** Caudal to the shunt the caudal vena cava is visible (asterisk)

**Fig. 2:**
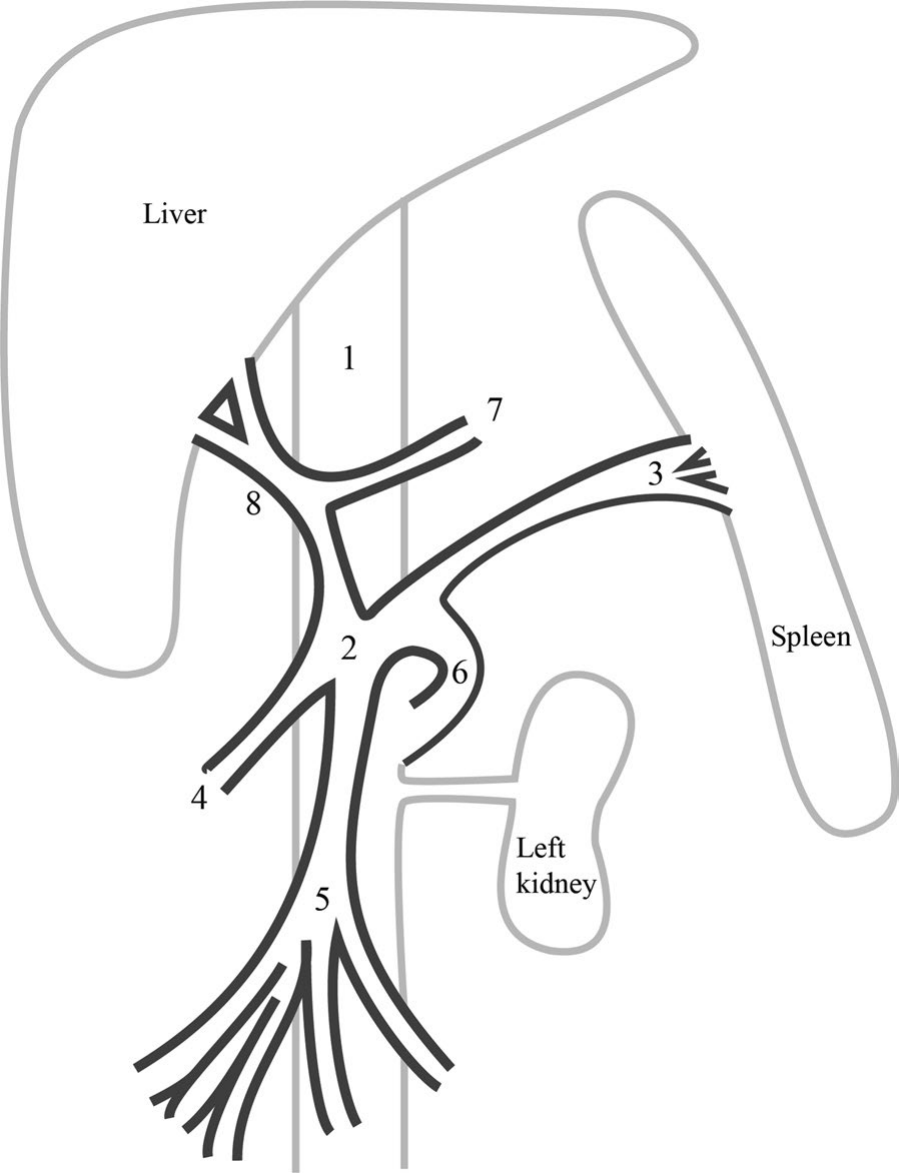
Illustration of the anatomy of the extrahepatic portocaval shunt. At the level of the junction of the splenic and portal vein, a shunting vessel loops to the left and caudally before merging with the caudal vena cava, just cranial to the left renal vein. 1. Caudal vena cava, 2. portal vein, 3. splenic vein, 4. caudal mesenteric veins, 5. cranial mesenteric veins, 6. splenocaval shunt, 7. gastroduodenal vein, 8. remaining portal branch to liver

CTA images confirmed the presence of an extra-hepatic portocaval shunt. A short abnormal vessel was identified, linking the portal vein with the caudal vena cava. At the junction of the splenic vein and portal vein, an abnormal vessel arose from the portal vein. The abnormal vessel, with a diameter of 2.5 cm, looped to the left and caudally, entering the left side of the caudal vena cava, just cranial to the left renal vein (Figs. 1 and 2). The total length of the shunt was approximately 5 cm. Cranial to the shunt, a well-developed portal vein with a diameter of 1.1 cm continued, which was joined by the gastroduodenal vein and then branched into the liver. The liver appeared normal in size and shape, with a homogenous attenuation before and after contrast medium administration.

Two days after confirmation of the diagnosis, surgery was initiated for partial occlusion of the shunt using a cellophane band. The foal was premedicated with 0.2 mg fentanyl (Fentadon) (see footnote 4) and 100 mg propofol (Propofol) (see footnote 6) IV, and anesthesia was induced with 300 mg propofol (Propofol) (see footnote 6) IV. The foal was intubated and maintained under general anesthesia by isoflurane inhalation (ET isoflurane 1.2%) and a constant rate infusion (CRI) of 0.25 mcg/kg/min fentanyl (Fentadon) (see footnote 4). Adequate blood pressure was maintained using dobutamine CRI (0.1–0.5 mcg/kg/min to effect, Dobutamine-hameln)^8^ and norepinephrine CRI (1.5–3 mcg/kg/min to effect, Noradrenaline CF).^9^ During surgery via midline celiotomy the caecum and colon were exteriorized as far as possible. The assumed portal vein and shunt were identified with great difficulty, due to their very dorsal localisation. Manual pressure on the colon, to keep it out of the surgical site, caused a small rupture of the colonic wall, with subsequent contamination of the abdomen. Given the likelihood that peritonitis would develop, the owner agreed to euthanasia.

## Discussion and conclusion

Congenital splenocaval extrahepatic shunting in foals has not been described frequently and not in such detail as in this case report. In dogs and cats portocaval shunts are more common and a hereditary cause is suspected in several breeds [6]. The morphology of different portocaval shunts in dogs and cats has been described in detail, and has led to a subdivision of portocaval shunts involving the splenic, left gastric, right gastric or colic vein [7–10]. According to this classification, the course of the abnormal vessel can be considered a case of splenocaval shunting.

Woodford et al. [3] described the course of an extra-hepatic shunt in a foal, determined using ultrasonography and intra-operative fluoroscopy. Similar to the current case, this shunt coursed caudally and was located in the region of the renal veins. However, the shunt described by Woodford et al. curved to the right instead of to the left as in the present case. The other published description of an extra-hepatic shunt in a foal was considered too limited to enable comparison with the current case [5].

A few of the previously described portosystemic shunts in foals were imaged by ultrasound or positive contrast portography (Table 1). In the current case no attempt was made to identify the shunt by ultrasound as CTA is considered superior for the detection of vascular anomalies and surgical planning [7–10]. Cross-sectional imaging with CTA provided a clear visualisation of the portosystemic shunt. Also, no attempt was made to visualize the shunt by ultrasound during surgery, since surrounding intestines, filled with gas and feces would have prevented the acquisition of clear images using this modality.

CTA of the portal system is routinely used to confirm a diagnosis of portosystemic shunts in dogs and cats. Different multi-phase computed tomography (CT) protocols have been described to investigate the portal system in companion animals [7–14]. Including the entire abdomen in the scan is recommended to allow identification of acquired extrahepatic shunts and to follow tortuous vessels [11]. In the current case, manual bolus injection of the contrast medium was chosen for practical reasons. Arterial and portal phase were not performed as described in multi-phase CTA in companion animals [7–14]. The timing of all phases was considered to be impossible at that moment because of size related scan duration and necessity for breath hold. Instead, manually started unenhanced, early venous phase and late venous phase scans were performed and provided detailed angiography of the portal veins and its tributaries.

A limitation of this study was the extent of the early venous phase of the CT scan. In this enhanced scan, the area from the diaphragm to the level of the middle of the right kidney was included. As the shunt joined the caudal vena cava caudal to the right renal vein, visualization of the shunt during the early venous phase scan was incomplete. The late venous phase scan did include the entire abdomen, but enhancement of vasculature was reduced compared to the early venous phase scan. Therefore, CTA of portosystemic shunts in foals should be performed of the entire abdomen or at least with inclusion of the kidneys, as portocaval shunts appear to occur in this area and therefore more caudally located than most types of small animal extra-hepatic portocaval shunts [7–14].

This is the first detailed description of CTA performed in a horse with an extra-hepatic splenocaval shunt. CTA provided a detailed overview of the abdominal and portal vasculature. When a portosystemic shunt is suspected in a foal, CTA represents a feasible technique for confirming the diagnosis and to allow surgical planning.

## Data Availability

All relevant data generated or analyzed during this study are included in this article and its supplementary information files.

## Abbreviations

CRI: constant rate infusion
CT: computed tomography
CTA: computed tomography angiography
IV: intravenous

## Footnotes

1 Equine hyperimmune plasma—Hypermune—Veterinary Immunogenics Ltd, Penrith, Cumbria, England.

2 Flunixin Meglumine 50 mg/g oral paste—Finadyne—Intervet, Boxmeer, The Netherlands.

3 Vitamin E 300 g—Equi-vitamin E—Equipharma—Ecuphar, Oostkamp, Belgium.

4 Fentanyl 0.05 mg/mL—Fentadon—Eurovet Animal Health BV, Bladel, The Netherlands.

5 Midazolam 5 mg/mL—Midazolam Actavis—Actavis Group PTC, Hafnarfjordur, Iceland.

6 Propofol 10 mg/mL—Propofol—Fresenius Kabi, Zeist, The Netherlands.

7 Xenetix 350 mg/mL—Iobitridiol—Guerbet Nederland BV, Gorinchem, The Netherlands.

8 Dobutamine 5 mg/mL—Dobutamine-hameln—Hameln pharma plus GmbH, Hameld, Germany.

9 Norepinephrine 1 mg/mL—Noradrenaline CF—Centrafarm BV, Etten-Leur, The Netherlands.
